# Serological cross-reactivity and identification of an acute Seoul orthohantavirus case in a dengue outbreak from Vietnam

**DOI:** 10.1093/trstmh/traf124

**Published:** 2025-11-18

**Authors:** Do Huy Loc, Do Duc Anh, Le Thi Kieu Linh, Do Thi Huyen Dieu, Ngo Thu Hang, Nguyen Huu Lanh, Dinh Thi Bich Thom, Nguyen Thi Hong Nhung, Peter G Kremsner, Jonas Schmidt-Chanasit, Le Huu Song, Nguyen Linh Toan, Thirumalaisamy P Velavan

**Affiliations:** Institute of Tropical Medicine, University of Tübingen and German Center for Infection Research (DZIF), Tübingen, Germany; Vietnamese-German Center for Medical Research (VG-CARE), Hanoi, Vietnam; Institute of Tropical Medicine, University of Tübingen and German Center for Infection Research (DZIF), Tübingen, Germany; Vietnamese-German Center for Medical Research (VG-CARE), Hanoi, Vietnam; Institute of Tropical Medicine, University of Tübingen and German Center for Infection Research (DZIF), Tübingen, Germany; Vietnamese-German Center for Medical Research (VG-CARE), Hanoi, Vietnam; Binh Dinh Medical College, Qui Nhon, Gia Lai, Vietnam; Department Pathophysiology, Vietnam Military Medical University, Hanoi, Vietnam; Binh Dinh Hospital, Qui Nhon, Gia Lai, Vietnam; Binh Dinh Hospital, Qui Nhon, Gia Lai, Vietnam; Binh Dinh Medical College, Qui Nhon, Gia Lai, Vietnam; Institute of Tropical Medicine, University of Tübingen and German Center for Infection Research (DZIF), Tübingen, Germany; Centre de Recherches Médicales de Lambaréné (CERMEL), Gabon; Bernhard Nocht Institute for Tropical Medicine, Hamburg, Germany; Faculty of Mathematics, Informatics and Natural Sciences, Universität Hamburg, Hamburg, Germany; Institute of Tropical Medicine, University of Tübingen and German Center for Infection Research (DZIF), Tübingen, Germany; Vietnamese-German Center for Medical Research (VG-CARE), Hanoi, Vietnam; 108 Military Central Hospital, Hanoi, Vietnam; Vietnamese-German Center for Medical Research (VG-CARE), Hanoi, Vietnam; Department Pathophysiology, Vietnam Military Medical University, Hanoi, Vietnam; Institute of Tropical Medicine, University of Tübingen and German Center for Infection Research (DZIF), Tübingen, Germany; Vietnamese-German Center for Medical Research (VG-CARE), Hanoi, Vietnam; Faculty of Medicine, Duy Tan University, Da Nang, Vietnam

**Keywords:** cross-reaction, dengue virus, flavivirus, hantavirus, Seoul virus, viral haemorrhagic fever

## Abstract

**Background:**

In dengue-endemic regions like Vietnam, viral haemorrhagic fevers (VHFs) with overlapping clinical presentations often lead to misdiagnoses. This study investigated alternative VHF pathogens in patients clinically suspected of dengue but negative by standard diagnostics during a 2016 outbreak in central Vietnam.

**Methods:**

Among 198 hospitalized patients, 52 dengue virus (DENV)-negative cases were tested using enzyme-linked immunosorbent assays for immunoglobulin G (IgG)/IgM against seven VHFs and molecular assays for pan-flaviviruses and hantaviruses. Positive amplicons were sequenced and subjected to phylogenetic analysis.

**Results:**

High flavivirus IgG seropositivity was observed, especially for Japanese encephalitis virus (96%), DENV, tick-borne encephalitis virus and West Nile virus (92% each), with notable cross-reactivity. IgM responses were specific, with 37% of patients positive for anti-JEV and 33% for anti-DENV. Pan-flavivirus reverse transcription polymerase chain reaction confirmed DENV-1 RNA in four patients. One patient tested positive for hantaviral RNA and seropositive for both IgG and IgM. Sequencing and phylogenetic analysis identified *Orthohantavirus seoulense* (SEOV) as the causative agent, clustering with SEOV strains from Vietnam and Indonesia. The detection of SEOV highlights co-circulation of rodent-borne viruses during arboviral outbreaks.

**Conclusions:**

This study highlights significant serological cross-reactivity among flaviviruses and underdiagnosed circulation of hantaviruses such as SEOV. Enhanced multimodal diagnostic surveillance of pathogens remains essential.

## Introduction

Viral haemorrhagic fevers (VHFs), caused by various arboviral pathogens, lead to high fever accompanied by bleeding complications. These VHFs represent a significant health threat, particularly in tropical regions, and place a heavy burden on healthcare systems, especially in sub-Saharan Africa and Southeast Asia.^1,2^ VHFs are caused by arboviruses and rodent-borne viruses, which are transmitted by arthropod vectors like mosquitoes and ticks, as well as by rodents such as mice and rats. Major VHF-causing viruses include dengue virus (DENV), Zika virus (ZIKV), chikungunya virus (CHIKV), Lassa virus (LASV) and hantaviruses such as Seoul virus (SEOV), which significantly impact the disease landscape in Africa and Asia.^3,4^

In Southeast Asia, where dengue fever is endemic, the clinical manifestations and symptoms overlap with other VHF infections and pose a significant challenge for accurate diagnosis, increasing the risk of misdiagnosis.^5,6^ To address these challenges, diagnostic algorithms typically rely on either molecular diagnostics or serological assays. Molecular diagnostics, particularly nucleic acid testing, are considered the gold standard for detecting viral RNA during the acute phase of infection.^7^ However, serological assays, such as enzyme-linked immunosorbent assays (ELISAs) and rapid diagnostic tests (RDTs), are more commonly used in resource-limited settings where the gold standard is often absent.^8^ While serology-based tests are more accessible in resource-limited settings, these methods often have lower specificity, particularly due to potential cross-reactivity with other VHF viruses circulating in the region.^5^ The combination of molecular and serological diagnostic tools improves overall diagnostic accuracy, particularly in regions where multiple viruses co-circulate.^9,10^

Vietnam is known for recurrent dengue outbreaks, with fluctuating serotypes over time and across regions.^11^ The aim of this study was to identify causative VHF pathogens in patients who tested negative for dengue using both serological and molecular diagnostic methods during an outbreak in central Vietnam in 2016. Because VHF caused by DENV and haemorrhagic fever with renal syndrome (HFRS) due to hantaviruses such as SEOV share overlapping clinical features, including fever, thrombocytopenia and potential haemorrhage, samples were screened not only for mosquito-borne viruses such as ZIKV and CHIKV using molecular assays but also for hantaviruses, alongside pan-flavivirus polymerase chain reactions (PCRs) to detect other flaviviruses. In addition, ELISA screening (immunoglobulin G [IgG] and IgM) was performed for a broad spectrum of seven VHF pathogens, including DENV (*Orthoflavivirus dengue*), ZIKV (*Orthoflavivirus zikaense*), CHIKV (*Alphavirus chikungunya*), Japanese encephalitis virus (JEV; *Orthoflavivirus japonicum*), West Nile virus (WNV; *Orthoflavivirus nilense*), tick-borne encephalitis virus (TBEV; *Orthoflavivirus encephalitidis*) and several hantavirus species: Hantaan virus (HNTV; *Orthohantavirus hantanense*), Dobrava virus (DOBV; *Orthohantavirus dobravaense*) and Puumala virus (PUUV; *Orthohantavirus puumalaense*).

## Methods

### Study population

The study included 198 hospitalised patients from Binh Dinh Central Hospital during the dengue outbreak between March and June 2016, all of whom were admitted with suspected dengue fever. Of the 198 patients, 146 were confirmed DENV positive (unpublished data), while the remaining 52 tested negative by both PCR and RDTs (NS1, IgG and IgM). In Vietnam, dengue diagnoses followed the World Health Organization (WHO) diagnostic criteria^12^ as adopted by the Vietnamese Ministry of Health. The diagnostic criteria were patients presenting with fever within 7 d of onset, accompanied by at least two clinical signs or symptoms suggestive of dengue (e.g. nausea/vomiting, rash, body aches and pains, tourniquet test positive) and positive for at least one of the indirect diagnostic methods (serological rapid test), as recommended and detailed in the WHO guideline 2009.^12^

Upon admission, laboratory parameters were recorded, including white blood cell (WBC) count, red blood cell (RBC) count, haemoglobin (Hb), haematocrit (HCT), platelet (PLT) count, urea, creatinine, aspartate aminotransferase (AST), alanine aminotransferase (ALT) and high-sensitivity C-reactive protein (hsCRP). Plasma samples were separated from blood and stored at −70°C for further analysis.

### Serological assays

The IgG and IgM antibodies for seven VHF pathogens (DENV, JEV, ZIKV, CHIKV, WNV, TBEV and hantaviruses) were measured using commercially available ELISA kits (Euroimmun, Lübeck, Germany). This included anti-dengue virus type 1–4 ELISA (IgG/IgM), anti-JEV ELISA (IgG/IgM), anti-Zika virus ELISA (IgG/IgM), anti-CHIKV ELISA (IgG/IgM), anti-WNV ELISA (IgG/IgM), anti-TBEV ELISA 2.0 (IgG/IgM) and anti-hantavirus pool 1 ‘Eurasia’ ELISA (IgG/IgM). The anti-hantavirus ELISA kit provides determination of IgG/IgM antibodies against three predominant hantavirus species: HTNV, DOBV and PUUV. As the ELISA kit detects IgG/IgM against HTNV, DOBV and PUUV, cross-reactivity within the hantavirus pool, including SEOV, may occur.^13^ Thus anti-hantavirus IgM positivity could reflect exposure to any of these viruses; confirmation through subsequent PCR identification of SEOV was performed. All assays were performed in duplicate following the manufacturer’s instructions. Briefly, plasma samples were diluted at a ratio of 1:101 and incubated at 37°C for 60 min. Samples were then incubated sequentially with the conjugate solution for 30 min and the substrate solution for 15 min at room temperature, with three washes performed between each step. The reaction was terminated using a stop solution and absorbance was measured at wavelengths of 450 and 620 nm with a CLARIOstar microplate reader (BMG Labtech, Ortenberg, Germany). Results were interpreted according to the manufacturer-defined cut-off indices provided.

### Viral RNA molecular testing

Total genomic RNA was extracted from the plasma of 52 patient samples using the QIAamp Viral RNA Mini Kit (Qiagen, Hilden, Germany) according to the manufacturer’s instructions. All 52 samples were initially analysed by multiplex real-time reverse transcription PCR (RT-PCR; Fast Track Diagnostics kit; Siemens Healthcare, Erlangen, Germany) for detection of DENV, ZIKV and CHIKV RNA on a LightCycler 480 II system (Roche, Mannheim, Germany). Each sample was independently tested twice in duplicates. Subsequently, all 52 samples were subjected to pan-flavivirus detection using RT-PCR using the SuperScript III One-Step RT-PCR System (Thermo Fisher Scientific, Waltham, MA, USA). The non-structural protein 5 (NS5) partial gene was amplified using pan-flavivirus primer pairs mFU1 (5′-TACAACATGATGGGAAAGCGAGAGAAAAA-3′) and CFD2 (5′-GTGTCCCAGCCGGCGGTGTCATCAGC-3′), using amplification conditions as described previously.^14^ A nested pan-hantavirus RT-PCR targeting a conserved region of the L-segment was also conducted on all 52 samples as previously described.^15^ The primer pairs used were as follows: outer forward: HAN-L-F1: (5′-ATGTAYGTBAGTGCWGATGC-3′) and outer reverse: HAN-L-R1 (5′-AACCADTCWGTYCCRTCATC-3′), and inner forward: HAN-L-F2 (5′-TGCWGATGCHACIAARTGGTC-3′) and inner reverse: HAN-L-R2 (5′-GCRTCRTCWGARTGRTGDGCAA-3′). PCR amplicons (approximately 380 bp) were visualised on a 1.5% agarose gel and then purified with Exo-SAP-IT (Applied Biosystems, Beverly, MA, USA) and sequenced with the BigDye Terminator version 1.1 Cycle Sequencing Kit (Thermo Fisher Scientific) on an ABI 3130XL DNA sequencer (Applied Biosystems).

### Phylogenetic analysis

Sanger sequences obtained in this study were analysed using Geneious Prime 2023.0.4 (Biomatters, Auckland, New Zealand) and compared against available sequences in the National Center for Biotechnology Information GenBank database. To assess the phylogenetic relationship of the hantavirus identified, a maximum likelihood phylogenetic tree was reconstructed using the best-fit substitution model (GTR+F+I+G4), based on representative reference sequences from diverse geographic regions. Sequence alignment was conducted using the MAFFT version 7.526 algorithm,^16^ and the phylogenetic tree was generated with IQ-TREE version 2.1.4,^17^ employing 1000 bootstrap replicates. The partial gene of the L-segment of the Hantaan virus sequence was submitted to GenBank under accession number PV933987.

### Statistical analysis

All data were analysed and visualised using R version 4.4.1 (R Foundation for Statistical Computing, Vienna, Austria).^18^ Categorical variables were summarized as counts and percentages while continuous variables were summarized as medians and interquartile ranges (IQRs). The normality of the distribution of quantitative variables was tested using the Shapiro–Wilk test. Categorical data were compared using the χ^2^ test while continuous variables were compared using the Student’s t‐test or Wilcoxon rank-sum test. Parametric data were analysed using Pearson correlation coefficients to assess relationships between IgG and IgM antibody titres for the viruses investigated. Data on the clinical presentations of the patients were unavailable for analysis.

## Results

### Demographic and laboratory characteristics of the study population

The study population had a median age of 35 y (IQR 26.5–49.5), with 54% being female. Alterations in laboratory parameters consistent with DENV infection were observed across the study cohort (Table 1). Notably, WBC counts were decreased (median 4.5×10^9^/l [IQR 3.2–6.65]) and PLT counts were markedly reduced (60×10^9^/l [IQR 19.8–99]), consistent with dengue-associated thrombocytopenia (Table 1). Additionally, liver enzymes were elevated, with median AST and ALT levels of 89 U/l (IQR 54.8–170) and 70.8 U/l (IQR 43.6–132.6), respectively. In addition, elevated hsCRP values (median 15.05 mg/l [IQR 1.75–45.2]) further indicated systemic inflammation in a substantial proportion of the study population (Table 1). No significant differences in laboratory parameters were observed between the 52 DENV-negative subjects and the 146 patients with confirmed dengue (Table 1). The patient confirmed as hantavirus-positive showed marked liver injury, with AST 349 U/l and ALT 161 U/l. Renal function was impaired (creatinine 159 µmol/l) and hsCRP peaked at 37 mg/l. Platelet count was profoundly reduced at 4×10^9^/l, while other haematological parameters, including WBC count (4.5×10^9^/l), Hb (158 g/l) and HCT (49.5%), remained within or near normal ranges.

**Table 1.** Comparison of baseline demographic and clinical characteristics between dengue-negative (n=52) and dengue-confirmed (n=146) patients in Gia Lai, 2016.

Variables	DENV negative (n=52)	DENV-confirmed patients (n=146)	p-Value
Age (years)	35 (27–50)	29 (19–41)	0.003
Female, n (%)	28 (54)	76 (52)	0.824
WBC (×10^9^/l)	4.5 (3.2–6.65)	3.5 (2.3–5.1)	0.012
RBC (×10^12^/l)	4.61 (3.84–5.11)	4.81 (4.39–5.25)	0.08
Hb (g/l)	137 (123–149)	138 (128–149)	0.371
HCT (%)	41 (35.85–44.65)	41.5 (37.6–44.4)	0.424
PLT (×10^9^/l)	60 (19.8–99)	76.5 (37–104)	0.082
Urea (mmol/l)	4.29 (3.47–4.9)	3.71 (2.9–4.6)	0.07
Creatinine (µmol/l)	79.5 (67.25–94.75)	79 (66–93)	0.974
AST (U/l)	89 (54.8–170)	97.9 (54.55–149.92)	0.876
ALT (U/l)	70.8 (43.6–132.6)	59 (30.8–111.8)	0.176
hsCRP (mg/l)	15.05 (1.75–45.2)	7.3 (1.98–20.9)	0.13

Values are presented as median (IQR) unless stated otherwise.

The p-value was computed using χ^2^ or Wilcoxon rank-sum tests.

### IgG cross-reactivity and IgM specificity

**Figure 1.**
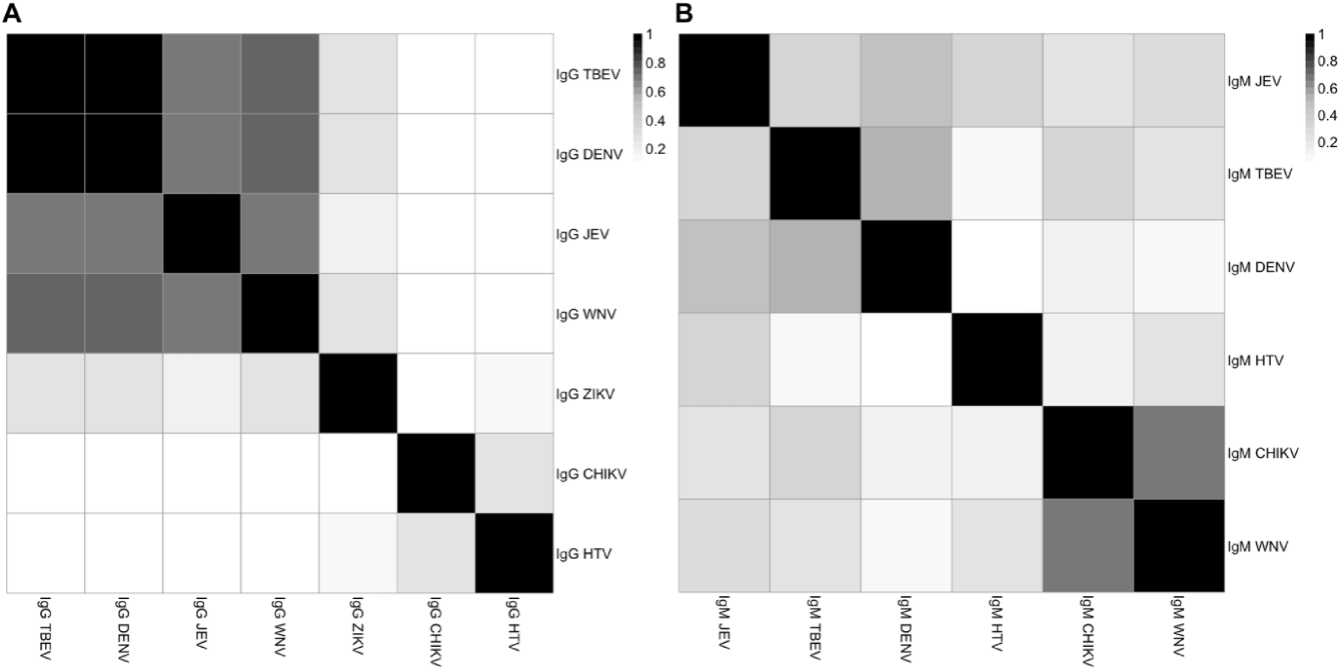
Correlation of **(A)** IgG and **(B)** IgM antibody responses to seven VHF-causing pathogens among dengue-negative patients (n=52) in Gia Lai, 2016. Variables represent correlation coefficients (r) between IgG and IgM responses. HTV: hantavirus.

IgG analysis revealed high seroprevalence for most flaviviruses: JEV (n=50/52 [96%]) and TBEV, WNV and DENV each showing similar positivity rates (n=48/52 [92%]) (Table 2). In contrast, anti-ZIKV IgG positivity was detected in only 46% of the cohort (n=24/52), while IgG positivity for hantaviruses and CHIKV remained relatively low, at 12% and 10%, respectively (Table 2). Significant correlations in IgG responses were observed, with strong co-positivity between DENV and TBEV (r = 1.00) and moderate correlations with WNV (r = 0.73) and JEV (r = 0.69), suggesting potential cross-reactivity among flaviviruses in these patients (Figure 1A, Supplementary Table S1).

**Table 2.** IgG and IgM seropositivity for other viruses among dengue-negative patients (n=52) in Gia Lai province, 2016.

Serology/pathogens	JEV	DENV	TBEV	WNV	ZIKV	Hantavirus	CHIKV
IgG, n (%)	50 (96)	48 (92)	48 (92)	48 (92)	24 (46)	6 (12)	5 (10)
IgM, n (%)	19 (37)	17 (33)	6 (12)	2 (4)	0 (0)	6 (12)	4 (8)

Variables are summarized as absolute counts (n) with percentages (%).

IgM responses were predominantly directed against JEV and DENV, with minimal or no reactivity to the other pathogens tested (Table 2). Anti-JEV IgM was detected in 19/52 patients (37%), followed by anti-DENV IgM in 17/52 patients (33%), suggesting recent circulation of these viruses (Table 2). Notably, 10/52 patients demonstrated monospecific IgM responses, consistent with probable acute infection by a single virus. These comprised four cases each of anti-JEV and anti-DENV IgM positivity and one case each of anti-CHIKV and anti-hantavirus IgM (Supplementary Table S2). In contrast, IgM positivity rates for TBEV, hantaviruses, CHIKV and WNV were low (≤12%) and no IgM reactivity was detected for ZIKV (Table 2). Correlations between IgM responses among flaviviruses were modest, with DENV-TBEV (r = 0.52) and DENV-JEV (r = 0.49) showing the highest values (Figure 1B, Supplementary Table S1).

### Viral RNA positivity

All 52 samples were initially tested using a multiplex real-time RT-PCR assay (fast track diagnostics kit) and were negative for DENV, ZIKV and CHIKV RNA. These samples also tested DENV negative using RDTs (NS1/IgG/IgM). To enhance viral detection, all 52 samples were subsequently screened using a pan-flavivirus PCR assay. Of these, four samples tested positive. Sanger sequencing of the corresponding PCR amplicons confirmed all four as DENV positive, specifically identified as dengue virus serotype 1 (DENV-1).

**Figure 2.**
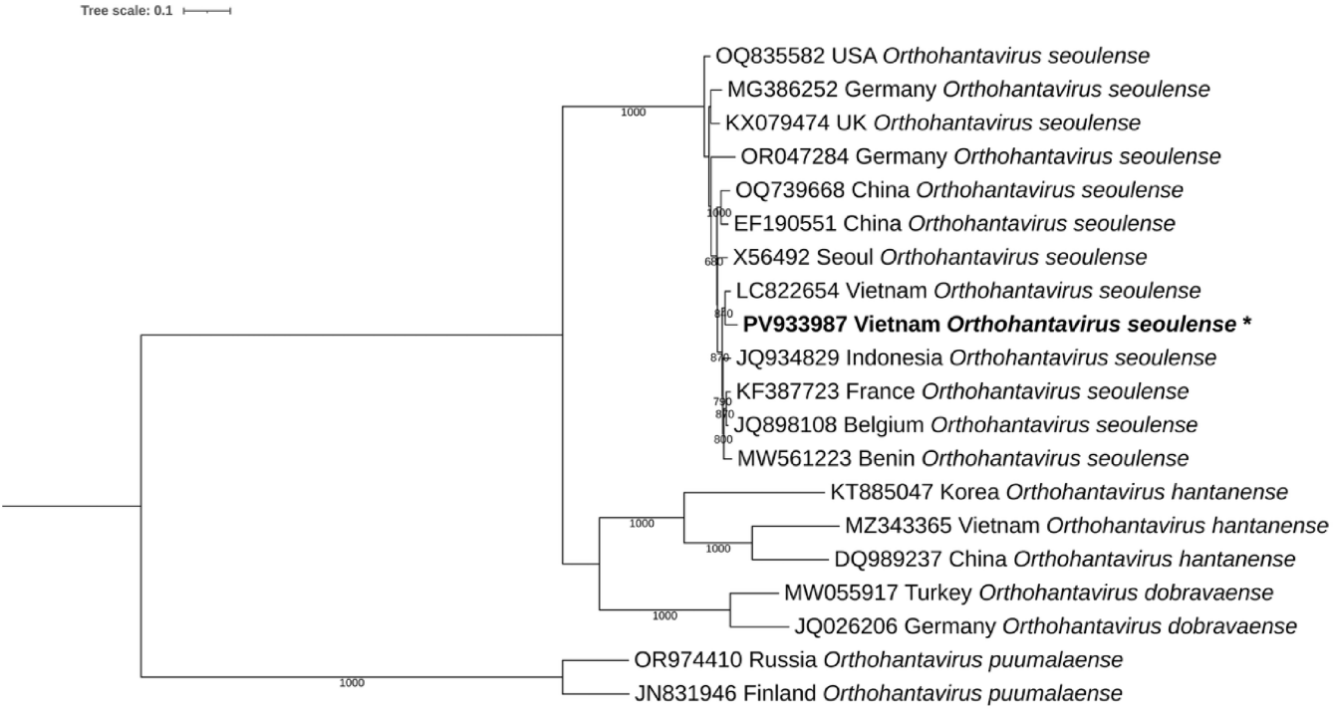
Maximum likelihood phylogenetic tree of a conserved region of the L segment of hantaviruses identified in a dengue-negative patient in Gia Lai, 2016. The phylogenetic tree was constructed using the GTR+F+I+G4 model with 1000 bootstrap replicates. The scale bar represents a sequence divergence of 0.1. Branch support values ≥60% are shown. The sequence obtained in this study is highlighted in bold and marked with an asterisk

Nested pan-hantavirus RT-PCR targeting a conserved region of the L-segment of hantaviruses detected one RNA-positive sample. This patient was tested twice independently to confirm the result. Phylogenetic analysis based on the partial viral genome sequence revealed that the sequence PV933987, obtained from the patient investigated in this study, clusters robustly within the species *Orthohantavirus seoulense* (SEOV) (bootstrap support: 840). It showed the closest genetic relationship to a Vietnamese SEOV sequence (LC822654) and clustered with globally distributed strains from Indonesia, Europe and Africa. This suggests that the sequence derived from the patient sample is part of a widely circulating lineage of *O. seoulense* (Figure 2).

## Discussion

This study highlights the diagnostic challenges posed by VHFs in regions such as central Vietnam, where multiple arboviruses and rodent-borne viruses circulate simultaneously. Although the initial clinical diagnosis during a known outbreak focused on dengue fever, our comprehensive serological and molecular tests identified a broader viral picture, including cases of SEOV and DENV-1, which had initially tested negative using routine diagnostic methods.

In 2016, Gia Lai province faced a significant dengue outbreak at the same time that Vietnam reported its first Zika cases, creating a unique setting to explore issues of arboviral co-circulation and diagnosis.^19,20^ Our findings demonstrate a high degree of flavivirus IgG seropositivity among the study population, with >90% of patients testing positive for antibodies against DENV, JEV, TBEV and WNV. This widespread seroprevalence may reflect historical exposure in an endemic setting and suggests significant immunological cross-reactivity within the flavivirus genus. The strong correlation between IgG responses to DENV, TBEV, WNV and JEV supports previous reports of serological cross-reactivity among these viruses.^21,22^ While this complicates serological interpretation, it underscores the necessity for confirmatory molecular diagnostics, particularly in outbreak settings.

In contrast to the broadly reactive IgG results, IgM responses were more specific, with a smaller proportion of patients showing virus-specific responses. Notably, 37% and 33% of patients were IgM-positive for JEV and DENV, respectively, while no IgM reactivity was detected for ZIKV, and low IgM rates were observed for CHIKV, hantaviruses, TBEV and WNV. These patterns suggest that although multiple viruses may have circulated in the region, only a subset of patients experienced recent infections. Importantly, 10 patients displayed virus-specific IgM responses, indicating probable acute or recent VHF infections beyond dengue.

Molecular screening confirmed the presence of DENV RNA in four samples using a pan-flavivirus RT-PCR assay, all subsequently typed as DENV-1. These cases had previously tested negative for DENV by both RDTs and a multiplex real-time RT-PCR assay, emphasising the limitations of single-platform diagnostics and the need for complementary approaches, particularly during the late acute or early convalescent phases when viral load may be low. Unlike real-time RT-PCR assays that target serotype-specific genomic regions, pan-flavivirus RT-PCR amplifies highly conserved regions, such as the NS5 gene, thereby enhancing the likelihood of detecting genetically divergent or low-titre dengue viruses.^23^

Notably, one patient in this study tested RNA positive for hantavirus by nested RT-PCR targeting the conserved L-segment. This patient exhibited marked liver injury, renal impairment, severe thrombocytopenia and elevated hsCRP. These abnormalities, particularly the renal dysfunction, profound thrombocytopenia and heightened inflammatory response, are characteristic of HFRS and highlight the need for vigilance in distinguishing hantavirus infection from dengue and other viral febrile illnesses. Although exposure history was not available, the clinical and laboratory findings strongly support hantavirus as the cause of illness in this case. While only a single case was confirmed, it provides sentinel evidence of hantavirus circulation during a dengue outbreak and underscores the importance of systematic surveillance to determine the true burden and epidemiological significance of hantaviruses in Vietnam.

Phylogenetic analysis identified the sample as SEOV, clustering closely with a previously reported SEOV strain from Vung Tau province in southern Vietnam, detected in 2024,^24^ and forming a well-supported clade with SEOV strains from Indonesia. Earlier studies have also shown that SEOV sequences identified in northern Vietnam form a single lineage with the Indonesian SEOV clade.^25^ Moreover, SEOV circulation has been reported among *Rattus norvegicus* populations in urban and harbour areas of southern Vietnam, reinforcing the role of this rodent host in virus maintenance and transmission.^26^ These findings indicate that SEOV is likely circulating throughout Vietnam and is part of a broader Asian dispersed lineage, possibly facilitated by the widespread distribution of its commensal rodent reservoir *R. norvegicus*. The patient in this study not only showed molecular evidence of SEOV infection but also tested positive for both IgG and IgM antibodies against hantaviruses, confirming acute infection.

SEOV infection can manifest as non-specific febrile illness and HFRS, which may be easily misdiagnosed as dengue fever in endemic regions lacking specific diagnostic tools. Notably, a confirmed case of HFRS due to SEOV was previously reported in southern Vietnam in 2008,^27^ underscoring the need for heightened clinical awareness and expanded diagnostic surveillance for hantaviruses in the region. This study reinforces the importance of incorporating hantavirus screening into differential diagnostics for suspected dengue cases, particularly when routine tests yield negative results. Furthermore, the identification of SEOV in a hospitalized patient during a dengue outbreak raises questions about co-circulation, rodent exposure risk, and underdiagnosed cases of hantavirus infection.

Limitations of our study include the restricted sample size and the focus on a single outbreak season. Additionally, while ELISAs provided valuable sero-epidemiological insights, their interpretation is limited by potential cross-reactivity. The detection of only one hantavirus RNA-positive case may underestimate true prevalence, given the short viraemic window and potential renal tropism of hantaviruses.

This study highlights the complexity of diagnosing VHFs in regions such as central Vietnam, where multiple arboviruses and rodent-borne viruses circulate simultaneously. The detection of DENV-1 in cases that initially tested negative in routine diagnostics highlights the limitations of single-platform testing and the value of molecular approaches for all flaviviruses. In particular, the identification of SEOV in a febrile patient confirmed by molecular and serological methods shows that rodent-borne hantaviruses are likely to be underdiagnosed and more widespread in Vietnam than previously thought. These findings highlight the need for integrated diagnostics and enhanced surveillance of non-dengue VHFs, particularly hantaviruses, to improve detection and public health response in Vietnam and beyond.

## Supplementary Material

traf124_Supplemental_File

## Data Availability

All data are available in the manuscript and the supplementary files. The partial L-segment sequence of the SEOV-positive sample from this study was submitted to GenBank with accession number PV933987.
