# Supplementary material for: Serological cross-reactivity and identification of an acute Seoul orthohantavirus case in a dengue outbreak from Vietnam
Source: Trans R Soc Trop Med Hyg. 2025 Nov 18;120(3):240–6. doi: 10.1093/trstmh/traf124 (PMC13017934; doi:10.1093/trstmh/traf124)
Supplement: traf124_Supplemental_File [file traf124_supplemental_file.docx]

**Supplementary Files**

**Table S1. Correlation of serological positivity between VHF-associated pathogens**

| IgG | | | | | | | |
| --- | --- | --- | --- | --- | --- | --- | --- |
|  | JEV | ZIKV | CHIKV | TBEV | Hantavirus | WNV | DENV |
| JEV | 1.00 | 0.19 | 0.07 | 0.69 | 0.07 | 0.69 | 0.69 |
| ZIKV | 0.19 | 1.00 | 0.09 | 0.27 | 0.15 | 0.27 | 0.27 |
| CHIKV | 0.07 | 0.09 | 1.00 | 0.09 | 0.29 | 0.09 | 0.09 |
| TBEV | 0.69 | 0.27 | 0.09 | 1.00 | 0.10 | 0.73 | 1.00 |
| HTV | 0.07 | 0.15 | 0.29 | 0.10 | 1.00 | 0.10 | 0.10 |
| WNV | 0.69 | 0.27 | 0.09 | 0.73 | 0.10 | 1.00 | 0.73 |
| DENV | 0.69 | 0.27 | 0.09 | 1.00 | 0.10 | 0.73 | 1.00 |
| IgM | | | | | | | |
|  | JEV | ZIKV | CHIKV | TBEV | Hantavirus | WNV | DENV |
| JEV | 1.00 | NA | 0.23 | 0.35 | 0.35 | 0.26 | 0.49 |
| ZIKV | NA | NA | NA | NA | NA | NA | NA |
| CHIKV | 0.23 | NA | 1.00 | 0.35 | 0.12 | 0.69 | 0.11 |
| TBEV | 0.35 | NA | 0.35 | 1.00 | 0.06 | 0.24 | 0.52 |
| HTV | 0.35 | NA | 0.12 | 0.06 | 1.00 | 0.24 | 0.00 |
| WNV | 0.26 | NA | 0.69 | 0.24 | 0.24 | 1.00 | 0.07 |
| DENV | 0.49 | NA | 0.11 | 0.52 | 0.00 | 0.07 | 1.00 |

Abbreviations: IgG: immunoglobulin G; IgM: immunoglobulin M; DENV: Dengue virus; JEV: Japanese encephalitis virus; ZIKV: Zika virus; CHIKV: Chikungunya virus; TBEV: Tick-borne encephalitis virus; WNV: West Nile virus; NA: not applicable.

**Table S2. IgM positivity among dengue negative patients**

| **IgM positivity for different viruses** | **n (%)** |
| --- | --- |
| DENV + JEV + CHIKV + TBEV + WNV | 1 (2) |
| DENV + JEV + CHIKV + TBEV | 1 (2) |
| DENV + JEV + Hantavirus + TBEV | 1 (2) |
| JEV + CHIKV + Hantavirus + WNV | 1 (2) |
| DENV + JEV + TBEV | 2 (4) |
| DENV + JEV + Hantavirus | 1 (2) |
| DENV + JEV | 6 (12) |
| DENV + TBEV | 1 (2) |
| JEV + Hantavirus | 2 (4) |
| DENV | 4 (8) |
| JEV | 4 (8) |
| CHIKV | 1 (2) |
| Hantavirus | 1 (2) |
| None | 26 (50) |
| **Total** | **52** |

Abbreviations: IgM: immunoglobulin M; DENV: Dengue virus; JEV: Japanese encephalitis virus; CHIKV: Chikungunya virus; TBEV: Tick-borne encephalitis virus; WNV: West Nile virus.
